# Transport, mechanical and global migration data of multilayer copolyamide nanocomposite films with different layouts

**DOI:** 10.1016/j.dib.2017.04.025

**Published:** 2017-04-27

**Authors:** P. Scarfato, E. Garofalo, L. Di Maio, L. Incarnato

**Affiliations:** Department of Industrial Engineering, University of Salerno, Via Giovanni Paolo II n. 132, 84084 Fisciano, SA, Italy

**Keywords:** Multilayer films, Polyamide nanocomposites, Co-extrusion, Transport properties

## Abstract

Transport, mechanical and global migration data concern multilayer food packaging films with different layouts, all incorporating a layered silicate/polyamide nanocomposite as oxygen barrier layer, and a low-density polyethylene (LDPE) as moisture resistant layer in direct contact with food. The data are related to “Tuning of co-extrusion processing conditions and film layout to optimize the performances of PA/PE multilayer nanocomposite films for food packaging” by Garofalo et al. (2017) [1]. Nanocomposite multilayer films, with different relative layer thicknesses and clay types, were produced using a laboratory scale co-extrusion blown-film equipment and were analyzed in terms of transport to oxygen and water vapor, mechanical properties and overall migration. The results have shown that all the multilayer hybrid films, based on the copolyamide layer filled with Cloisite 30B, displayed the most significant oxygen barrier improvements and the best mechanical properties compared to the unfilled films. No significant alteration of the overall migration values was observed, as expectable [2], [3], [4]. The performance improvement was more relevant in the case of the film with the thinner nanocomposite layer.

**Specifications Table**

**Table:** 

Subject area	*Engineering*
More specific subject area	*Material Technology*
Type of data	Supplementary material
How data was acquired	*GPC-D Gas Permeability Tester (Brugger, Germany), Water Vapor Permeation Analyzer Model 7002 (Systech Illinois, UK) and CMT4000 SANS Series dynamometer (by MTS, China).*
Data format	*Excel 2010*
Experimental factors	*Nanocomposite multilayer packaging films with different layer composition and relative layer thicknesses were produced using a laboratory scale co-extrusion blown-film equipment. The multilayer films incorporate a polyamide/clay nanocomposite layer, acting as oxygen barrier, and a low-density polyethylene layer, acting as moisture barrier, coupled together by an adhesive tie-layer. The polyamide/clay nanocomposite layer was composed of a copolyamide 6,66 (CS40LXW, Radici Groups) melt compounded with two different commercial organomodified layered silicates, Cloisite 30B or Dellite 43B, at 4 wt% loading.*
Experimental features	*The performances of the nanocomposite multilayer films were related to the system layout.*
Data source location	*Department of Industrial Engineering, University of Salerno, Fisciano (SA), Italy*
Data accessibility	*Data are available with this article*

**Value of the data**

• In the literature few experimental data on transport and mechanical properties of multilayer nanocomposite films are available.
• The experimental data demonstrate the key role of the multilayer film layout in the film performances.
• The data allow to identify film configurations able to maximize O_2_ and water vapor barrier and stiffness.
• The provided data can be used as input properties for validation or calibration of analytical models.

## 1 Data

Experimental details are described in Ref. [1]. The data presented here are related to three-layer films (PA/tie/PE) with different layouts and composition, as specified in Table 1. In these data, the effects of relative layer thickness and clay type on transport and mechanical properties of the films were investigated.

The oxygen transmission rates (OTR) of the neat and nanocomposite multilayer structures with different film layouts are reported in the Excel file denoted as “oxygen permeability data”.

With respect to the corresponding unfilled systems produced, all the C30B and D43B-based nanocomposite multilayer films show a significant decrease in their OTR values, i.e. a significant increment of their oxygen barrier properties, ranging in the 50–60% interval. Moreover, in the case of the unfilled systems, the OTR values increase with decreasing the thickness of the oxygen barrier PA layer and with increasing the film stretching conditions from type S1 to type S3 structure. Instead, for the hybrid multilayer films the OTR values remain essentially unchanged passing from type S1 to type S2 structures and increase passing from type S2 to S3 structures.

The water vapor transmission rates (WVTR) of the multilayer films as a function of film layout are reported in the Excel file denoted as “water vapor permeability data”. The WVTR values resulted on average higher for the systems with a thicker layer of polyamide (S1-0, S1-C and S1-D), due to the hydrophilic nature of this latter polymer. However, both nanofillers slightly reduced the WVTR data of the hybrid films compared to the corresponding unfilled structures. This barrier improvement was more relevant (20%) in the samples characterized by a thicker polyamide layer (S1-C and S1-D). In particular, the lowest WVTR values were obtained when D43B was incorporated in the copolyamide matrix (S1-D and S2-D) due to higher degree of hydrophobicity of this organically modified nanoclay compared to C30B. As expected, the reduction of the PE layer from type S2 to type S3 structures determined an increase of the WVTR values in all the systems.

The tensile mechanical properties of the unfilled and nanocomposite multilayer structures are reported in the Excel file denoted as “mechanical data”. The presence of both the organoclays determined interesting increments in the stiffness (55%) and tensile strength (50%) of the multilayer films. In particular, the most significant improvements were obtained in the samples with C30B. The stiffness increments of the hybrid films, respect to the corresponding unfilled systems, are higher in type S2 structures, co-extruded at higher draw ratio (and thus having lower polyamide layer thickness), than in the type S1 ones. Moreover, no significant reduction in the ductility of all the hybrid multilayer films can be observed compared to the neat multilayer structures.

**Fig. 1:**
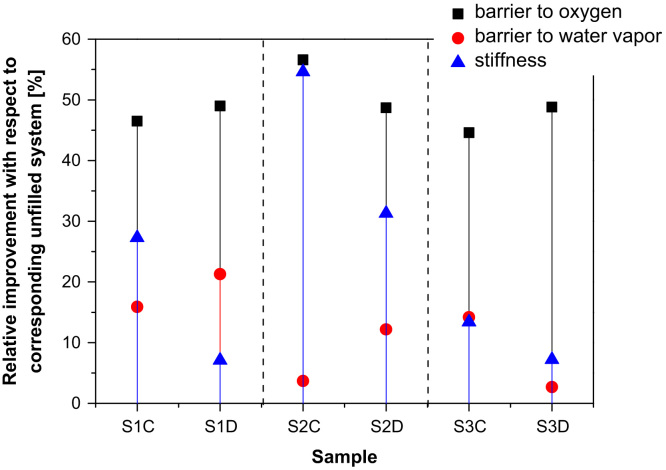
Graph summarizing the barrier (to oxygen and water vapor) and stiffness increments for the nanocomposite systems respect to the corresponding neat multilayer films. Reprinted and modified with permission from [1].

A comprehensive picture of the effects of both the organoclay type and processing conditions on the multilayer films’ performances, the increments in oxygen and water vapor barrier as well as stiffness increments for the nanocomposite films, respect to the corresponding neat systems, is shown in Fig. 1. The graph evidences that the coextruded films with the thinner nanocomposite layer (S2-C and S2-D) and, in particular, the system filled with C30B, display the most significant increment in stiffness and oxygen barrier properties, whereas the multilayer films with D43B (S1-D and S2-D) show the most interesting water vapor barrier performances.

Table 2 reports the overall migration values in rectified olive oil (CEN method EN 1186-4:2002) of selected multilayer films.

The overall migration data obtained for the multilayer films tested in this work were always significantly lower than the legislation limit of 10 mg/dm^2^ [2], [3], [4].

## 2 Experimental design, materials and methods

### 2.1 Materials

All materials were selected according to the results of our previous studies on polyamide/clay nanocomposite systems [5], [6], [7], [8], [9], [10]. Two montmorillonites, organically modified with different ammonium salts, were used as nanofillers: Cloisite 30B (C30B, by Southern Clay Products, Inc.), modified by methyl,tallow,bis-2-hydroxyethyl quaternary ammonium chloride, has a basal interlayer spacing *d*_001_=18.5 Å, and Dellite 43B (D43B, by Laviosa Chimica Mineraria S.p.A.), modified by dimethyl,benzyl,hydrogenated tallow ammonium salt, has a basal interlayer spacing *d*_001_=18.6 Å. The polymeric materials used in the coextruded blown films were a copolyamide 6,66 (CS40LXW, by Radici Group), referred to as PA, and a linear low density polyethylene (Riblene FL30, supplied by Versalis S.p.A.), referred to as PE. Moreover, an adhesive linear low density polyethylene grafted by maleic anhydride (LDPE-g-MAH ADMER NF358E, by Mitsui Chemicals), having good affinity with both the polyamide and the polyethylene resins, was used as tie layer.

The PA nanocomposites at 4 wt% of organoclay were prepared by melt compounding using a co-rotating intermeshing twin-screw extruder (dr. Collin GmBH model ZK 25-48D) with screws diameter (D) of 25 mm and length of 42 D. The extrusion temperature was set at 250–245–230 °C along the barrel and 230 °C at the die and the extrusion speed was 220 rpm. Extruded materials were cooled into a water bath and pelletized. Due to their hydrophilic nature, the PA and the clays were dried in a vacuum oven for 18 h at 90 °C before processing.

### 2.2 Multilayer film production

The multilayer films were produced by means of a laboratory coextrusion blown film apparatus, equipped with three single screw extruders (*D*_screw_=12 mm, *L*/*D*=24), a 3-layer blow film die (Collin, Type RWT 25, *D*_in_=30 mm, *D*_ext_=32 mm) and a take-up/cooling system (Collin Film Blowing Line Type BL 50). Three different types of films, referred as S1, S2 and S3, were prepared feeding the materials separately into their individual extruders heated according to the following thermal profiles: Extruder 1 (Tie-layer): 230–220–220–235 °C; Extruder 2 (PA): 245–245–245–235 °C; Extruder 3 (PE): 230–220–220–235 °C.

The extruders were operated at different screw speeds, in order to modify the mass flow rate of the output materials and thus the relative layer thicknesses and the draw ratios of the multilayer films. The extruded bubble films were inflated and cooled with air, so to obtain a bubble blow-up ratio (BUR) equal to 2, and stretched by a take-up device that collected the films at a fixed collection speed of 3 m/min. Table 1 summarizes the nomenclature and the characteristics of the produced coextruded structures.

Due to their hydrophilic nature, both the neat PA, the nanocomposite PA blends and the LDPE-g-MAH were dried in a vacuum oven for 18 h at 90 °C before processing.

### 2.3 Film characterization

Oxygen permeability of the films was determined with GPC-D Brugger permeability device at 23 °C, according to ASTM D1434 procedure. The standard deviations of all measured OTR values were less than 10%.

Water vapor permeability tests were performed with a Water Vapor Permeation analyzer (Model 7002 – Systech Illinois) that uses a sensor of P_2_O_5_. The measurements were carried out on a circular area of 5 cm^2^ at 23 °C and at a relative humidity of 85%, according to the standard ASTM F 1249. The standard deviations of all measured WVTR values were less than 5%.

Film specimens were cut, along the extrusion direction, with a rectangular geometry of 12.7×80 mm^2^ and tested, according to the ASTM D882, by means of a CMT4000 SANS Series dynamometer (by MTS, China). A crosshead speed of 5 mm/min was set to measure Young׳s modulus and 500 mm/min to determine the stress and elongation at break.

Overall migration tests were conducted in triplicate on the multilayer films, according to the experimental procedure described in detail in the official CEN method EN 1186-4:2002. In particular, the PE layer of each coextruded film was kept in contact with rectified olive oil (simulant D2) at 40 °C for 10 days, using one-side contact migration cells, specifically designed for multilayer structures. Simulant D2 was selected as “worst-case” simulant for potential migration from polyethylene. All the single overall migration determinations were within the analytical tolerance of 3 mg/dm^2^ for fatty food simulants, allowed by Regulation EU 10/2011.

## Figures and Tables

**Table 1:** Nomenclature and description of the multilayer films produced with different layouts. Reprinted with permission from [1].

**Film type**	**Film nomenclature and layout**	**Extrusion speed [rpm]**	**Layer thicknesses [μm]**	**Total film thickness [μm]**	**Draw ratio**
	**S1-0: PA/tie/PE**	70/20/70	45/8/27		
**S1**	**S1-C: PA+C30B/tie/PE**			80±1	5
	**S1-D: PA+D43B/tie/PE**				
	**S2-0: PA/tie/PE**	45/20/70	35/8/27		
**S2**	**S2-C: PA+C30B/tie/PE**			70±3	6.5
	**S2-D: PA+D43B/tie/PE**				
	**S3-0: PA/tie/PE**	45/20/45	35/8/17		
**S3**	**S3-C: PA+C30B/tie/PE**			60±2	8
	**S3-D: PA+D43B/tie/PE**				

**Table 2:** Overall migration values of selected multilayer films after contact with rectified olive oil (simulant D2) at 40 °C for 10 days, using migration cells for one-side contact. Reprinted with permission from [1].

**Film type**	**Overall migration [mg/dm**^**2**^**]**		
	**Clay type**		
	**Neat**	**C30B**	**D34B**
**S1**	0.21±0.16	1.54±1.30	–
**S2**	0.33±0.22	0.25±0.19	0.28±0.20
**S3**	0.19±0.14	0.28±0.25	–
